# Palliative and end of life care in undergraduate medical education: a survey of New Zealand medical schools

**DOI:** 10.1186/s12909-022-03593-3

**Published:** 2022-07-08

**Authors:** Lis Heath, Richard Egan, Ella Iosua, Robert Walker, Jean Ross, Rod MacLeod

**Affiliations:** 1 Department of Medicine, University of Otago, Dunedin, New Zealand; 2 Department of Preventive and Social Medicine, University of Otago, Dunedin, New Zealand; 3 Biostatistics Centre, University of Otago, Dunedin, New Zealand; 4 School of Nursing, Otago Polytechnic, Dunedin, New Zealand; 5 School of Population Health, University of Auckland, Auckland, New Zealand

**Keywords:** Palliative, End of life care, Education, Undergraduate, Curriculum, Medical students

## Abstract

**Background:**

In New Zealand, 34% of deaths occur in the hospital setting where junior doctors are at the frontline of patient care. The death rate in New Zealand is expected to double by 2068 due to the aging population, but many studies report that graduates feel unprepared to care for people near the end of life and find this to be one of the most stressful parts of their work. International guidelines recommend that palliative and end of life care should be a mandatory component of undergraduate medical education, yet teaching varies widely and remains optional in many countries. Little is known about how medical students in New Zealand learn about this important area of clinical practice. The purpose of this study was to investigate the organisation, structure and provision of formal teaching, assessment and clinical learning opportunities in palliative and end of life care for undergraduate medical students in New Zealand.

**Methods:**

Quantitative descriptive, cross-sectional survey of module conveners in New Zealand medical schools.

**Results:**

Palliative and end of life care is included in undergraduate teaching in all medical schools. However, there are gaps in content, minimal formal assessment and limited contact with specialist palliative care services. Lack of teaching staff and pressure on curriculum time are the main barriers to further curriculum development.

**Conclusions:**

This article reports the findings of the first national survey of formal teaching, assessment and clinical learning opportunities in palliative and end of life care in undergraduate medical education in New Zealand. There has been significant progress towards integrating this content into the curriculum, although further development is needed to address barriers and maximise learning opportunities to ensure graduates are as well prepared as possible.

## Background

Palliative care and end of life care (PEOLC) is widely regarded as an important component of undergraduate medical education. However, this subject remains optional in many areas, which fails to prepare doctors for the inevitable reality that patients will die while in their care. The COVID-19 pandemic brought this issue into sharp relief as healthcare providers around the world have struggled to meet demand as hospitals became overwhelmed with sick and dying patients. While New Zealand (NZ) has fared better than most countries during the pandemic, the death rate from life-limiting diseases is expected to double by 2068 with most deaths occurring in people aged > 85 years. This increase will be reflected across all healthcare settings, with a 51% rise in palliative care need by 2038 [1]. These statistics reflect a global challenge in healthcare that is, unfortunately, not amenable to a vaccine.

Approximately one-third of deaths in NZ occur in acute care hospitals where at least 20% of inpatients meet the Gold Standards criteria for palliative care [2]. Most graduates complete their first 2 years of clinical practice in hospital settings so they are likely to encounter people with palliative and end of life care needs on a regular basis. In fact, a study from the United Kingdom (UK) which investigated medical students’ attitudes towards palliative care, reported that junior doctors care for approximately 40 people who die, and a further 120 people who are close to the end of life, in their first year after qualifying [3]. Given the NZ statistics previously mentioned, it seems likely that NZ graduates may experience a similar reality.

PEOLC has not traditionally been included in undergraduate teaching due to an emphasis on curative medicine and the availability of treatments and investigations which seek to prolong life at any cost. Unfortunately, this denies patients and their family or whānau (translated from Māori as extended family, which may include friends who have no kinship ties to other members, see:https://maoridictionary.co.nz) the care and support they need at a time when quality of life should be a priority. In the absence of such training, newly qualified doctors are likely to enter clinical practice without the necessary knowledge, skills or attitudes to provide basic palliative care. However, attitudes are changing and this content is now being integrated into undergraduate curricula around the world, which is a fundamental step towards improving awareness, workforce capacity and increasing access to primary (non-specialist) palliative care [4, 5].

The very nature of caring for people with life-limiting conditions is inherently challenging, and studies report that newly qualified doctors find caring for people toward the end of life stressful and emotionally distressing [3, 6–8]. This is perhaps, not surprising. However, deficiencies in undergraduate education such as lack of meaningful contact with people who are dying, lack of role modelling by senior physicians, curative oriented health systems and variable teaching, fails to prepare students for one of the most challenging aspects of their work. These issues ultimately compromise patient care and increase graduates’ anxiety at a critical point in their professional development [6, 9, 10]. Negative personal attitudes and beliefs about death and dying may also influence graduates’ willingness to care for people at the end of life [11], although education has been shown to be effective in addressing these issues [12, 13]. In the absence of such education, graduates must rely on their intuition and guidance from colleagues, which may be neither available nor appropriate if their colleagues have not received formal training in palliative and end of life care (PEOLC).

In 2014, a declaration by the World Health Assembly stated that “palliative care should be integrated as a routine component of undergraduate medical education” to improve access to primary palliative care [14]. International recommendations suggest teaching should be based on nationally agreed and developmentally appropriate competencies with dedicated clinical exposure, reflection and discussion about challenging cases, assessment and multidisciplinary input [15, 16]. A variety of international guidelines and competency frameworks have since been developed to support this work. For example, the EDUPALL curriculum, based on recommendations from the European Association for Palliative Care (EAPC) (edupall.eu); Educating Future Physicians in Palliative and End of Life Care curriculum in Canada (EFPPEC); Palliative Care for Undergraduates sponsored by the Australian government (pcc4u.org); All Ireland Institute of Hospice Palliative Care competence framework (http://aiihpc.org/), and the Northern Ireland Health and Social Care Palliative and End of Life Care Competency Assessment Tool.

Early efforts to include PEOLC in undergraduate curricula tended to be optional, fragmented and lacked coordination [10]. However, there is now greater consistency in curricular offerings [17]. Canada, the UK, Austria, Belgium, Estonia, Switzerland, Israel, Luxembourg, Moldova, Belgium, France, and Germany now include PEOLC as a mandatory component of undergraduate medical education. A further 13 European countries teach PEOLC combined with another medical specialty, e.g. palliative care and oncology [18–20]. Unfortunately, few European countries offer more than 20 hours of formal palliative care teaching or mandatory clinical experience in palliative care. In the US, medical schools are not required to teach palliative care competencies. While a 2014 review reported that palliative care is included in most medical schools’ curricula, teaching varies widely and is underdeveloped [16]. Similarly, a 2014 report described PEOLC teaching in Australian medical schools as variable, fragmented and disjointed. Despite global efforts to increase undergraduate PEOLC education, overcrowded curricula, insufficient time, lack of faculty expertise and leadership, and a lack of funding and assessment, thwart the ability to do so [16, 21–23]. Despite that, student evaluations show they value this teaching, consider it relevant to general clinical practice, and feel it improves confidence in their ability to care for people near the end of life [24]. Palliative care teaching has also been shown to foster holistic patient-centred care and professional development [25].

New Zealand is currently ranked third on the global Quality of Death Index, which rates the provision of palliative care worldwide based on income as a predictor of the availability and quality of services [26]. Specialist palliative care services are well established in urban centres throughout NZ, although people in rural and remote communities often have to travel long distances to access this care. Therefore, up to 80% of palliative care is provided by health professionals who may not have had any formal training in palliative care [1], supported by specialist palliative care services (where available), and the national guidelines: Te Ara Whakapiri - Principles and Guidance for The Last Days of Life [27]. Advanced vocational training to prepare for independent practice as a Palliative Medicine Consultant is available through the Royal Australasian College of Physicians (RACP) as a 3 year full time course with clinical supervision (https://www.racp.edu.au/trainees/advanced-training/advanced-training-programs/palliative-medicine). However, there is an impending shortage of palliative medicine specialists and district health boards report difficulty recruiting and retaining suitably qualified staff. This situation has led to inequities in access for patients and families/whānau needing care, and for healthcare providers seeking support [1]. These issues were highlighted in the NZ Ministry of Health Palliative Care Action Plan [28], which prioritised workforce development by *“supporting work to modify undergraduate education and training to provide the minimum knowledge and skills related to primary palliative care”* (p.23). This statement provides a clear mandate for medical schools to ensure PEOLC is comprehensively addressed in the undergraduate curriculum to ensure graduates have the necessary skills to provide equitable access to primary palliative care across all healthcare settings and geographic locations. Unfortunately, the shortage of palliative medicine specialists required to teach this material may undermine the ability to deliver on this priority.

There are two accredited providers of undergraduate medical education in NZ. These medical schools are located at the University of Otago (Dunedin) and the University of Auckland (Auckland), with a combined annual intake of around 550 students. Students gain entry through a highly competitive one-year health sciences course, or the alternate pathway for those with a prior degree, followed by 2 years of pre-clinical education with some early clinical exposure, and 3 years of clinical training in hospital and community settings. The sixth and final year is spent working as a Trainee Intern in hospital settings. Graduates obtain provisional registration and are awarded with a Bachelor of Medicine and Bachelor of Surgery degree (MBChB). They then undertake 2 years of prevocational training as a House Officer (PGY1–2) before being registered as a medical practitioner [29]. Medical students at Otago University complete the first 3 years of the course in Dunedin, then divide into thirds for years 4–6 in one of three clinical teaching campuses in Dunedin, Christchurch or Wellington. Medical students at Auckland University follow a similar pathway with clinical attachments in hospital and community settings throughout Auckland and regional centres in the North Island.

While MBChB programmes are accredited by the Australian Medical Council, the Medical Council of New Zealand [30] sets the standards for medical practice, which includes care at the end of life. Learning outcomes and objectives in PEOLC are included in each universities’ curriculum documents to influence curriculum planning, development delivery and assessment. There are no academic professors of palliative medicine at either of NZ’s medical schools and only a handful of academic appointments to teach palliative care.

In 1997, a palliative care curriculum for undergraduate medical students was introduced by the Australian & New Zealand Society for Palliative Medicine (ANZSPM) and accepted by the Deans of all medical schools in Australia and NZ but unfortunately, little change eventuated. However, collaboration between Otago and Auckland universities to address deficiencies in PEOLC teaching has since resulted in the development of a national undergraduate PEOLC curriculum and implementation framework, which incorporates the previous work done by ANZSPM. It is against this background that the first national survey of NZ medical schools was undertaken to provide a baseline for further development.

## Methods

This study used quantitative descriptive methodology with an anonymised, national, online cross-sectional survey of medical schools to obtain information about the organisation, structure and provision of formal teaching, assessment and clinical learning opportunities in PEOLC in undergraduate medical education in New Zealand. The study was approved by the University of Otago Human Ethics Committee on 31 January 2019 (ref #: 19/009).

The online survey was adapted (with permission) from a UK survey of medical schools [19], initially developed by Field and Wee [31], and delivered using the Qualtrics survey platform. Data collection occurred between March–June 2019.

### Setting and participants

Survey participants were module conveners responsible for coordinating formal PEOLC teaching, learning opportunities and assessment activities at each of the four main campuses, so they have a comprehensive understanding of the PEOLC curriculum. There is one convener at each campus (*n* = 4), all of whom participated (RR = 100%). Conveners were asked to select from a range of potential responses to questions about the organisation, leadership, curriculum content, teaching methods, clinical exposure to palliative care providers, and formal assessment in PEOLC at their campus. Space for free text comments allowed conveners to provide more detail with an assurance that all information would be reported anonymously. The survey was piloted by a group of educators and an e-learning facilitator before being distributed. No changes were required.

Each convener was contacted by email which contained a link to the survey. The survey was front-loaded with the information sheet and consent form, then directed participants to the survey, which took approximately 15 minutes to complete. Three reminders were sent at ten-day intervals before closing the survey. An initial report was obtained through the Qualtrics survey platform, and STATA software version 15.0 was used to calculate the descriptive statistics.

## Results

Replies were received from all four PEOLC module conveners (RR = 100%) from Otago and Auckland University medical schools located in Dunedin and Auckland, and the two University of Otago affiliated teaching sites (campuses) in Christchurch and Wellington.

### Organisation and leadership

An identified convener is responsible for coordinating PEOLC teaching on each campus. All conveners reported that PEOLC is vertically integrated throughout the undergraduate curriculum beginning in year 2. A total > 40 hours of formal teaching from years 2–6 was reported by three conveners (not including clinical attachments), and one convener reported 21–30 hours. Programme evaluation reflects a commitment to ongoing improvement and all conveners reported that PEOLC teaching is routinely evaluated. However, formal assessment of student learning in PEOLC was only reported by three conveners. All conveners receive financial remuneration for coordinating PEOLC education as part of their wider roles and responsiblities, have advanced training in palliative care and are clinically active in the specialty. The majority of teaching is provided by medical and nursing staff from either a palliative care or primary care background, with additional input from other disciplines such as counsellors, social workers, spiritual care coordinators and academic lecturers.

### Teaching methods

All conveners reported using lectures, tutorials and reflective activities for PEOLC teaching. Supervised clinical experience with palliative care providers is available on all campuses, albeit for a minimal time. Case discussions and DVDs, films and podcasts are used on three campuses. Interviews or presentations with patients and informal carers are used on two.

campuses, and one convener reported the use of E-learning resources.

### Teaching content and perceived adequacy

Table 1 presents the range of subjects included in PEOLC teaching at the time the survey was conducted, and to what extent these subjects were addressed in the curriculum. Three conveners reported that approximately half of these subjects are either adequately or comprehensively addressed. Subjects taught only ‘a little’ or ‘not covered’ at all included cultural and religious issues, palliative care emergencies, assessment and management of other symptoms, confusion and constipation. Only one convener reported that these subjects were ‘adequately’ or ‘comprehensively’ addressed in their curriculum. Communicating in high stakes situations was addressed ‘a little’ on three campuses, and only two conveners reported that spirituality, nutrition and hydration, last days of life, personal coping, and loss, grief & bereavement were ‘adequately’ or ‘comprehensively’ addressed. Teaching about ethical issues is covered to varying extents ranging from ‘a little’ to ‘comprehensive’ at three schools. Euthanasia and assisted dying will need to be carefully reviewed with the introduction of legislation in November 2021 that legalised assisted dying.

**Table 1:** Teaching content and perceived adequacy

	“Not covered” n (%)	“A little” n (%)	“Adequate” n (%)	“Comprehensive” n (%)
Definition & philosophy of palliative care	0	1 (25)	0	3 (75)
Attitudes towards death & dying	0	0	1(25)	3(75)
Social contexts of dying e.g. home, rest home, hospital, hospice	0	1(25)	2(50)	1(25)
Assessment & management of pain	0	0	1(25)	3(75)
Assessment & management of nausea & vomiting	0	1(25)	2(50)	1(25)
Assessment & management of breathlessness	0	1(25)	2(50	1(25)
Assessment & management of constipation	0	3(75)	0	1(25)
Assessment & management of agitation & confusion	1(25)	2(50)	1(25)	0
Assessment & management of other symptoms	1(25)	2(50)	1(25)	0
Non-pharmacological symptom management	0	1(25)	3(75)	0
Nutrition & hydration at the end of life	0	2(50)	2(50	0
Care in the last days of life	0	2(50)	2(50)	0
Palliative care emergencies	1(25)	2(50)	1(25)	0
Loss, grief & bereavement	1(25)	1(25)	1(25)	1(25)
Impact of illness on patient & family/whanau (e.g. anxiety & depression)	0	1(25)	1(25)	2(50)
Spirituality (e.g. the role of spirituality in healthcare, spiritual distress, hope)	1(25)	1(25)	1(25)	1(25)
Cultural & religious issues in end of life care	1(25)	2(50)	1(25)	0
Ethical & legal issues e.g. futility, withdrawing/withholding treatment, euthanasia)	0	1(25)	2(50)	1(25)
Mental capacity, advance directives & Enduring Power of Attorney	0	1(25)	2(50)	1(25)
Death certification	0	1(25)	3(75)	0
Communicating in high stakes situations (e.g. goals of care, advance care planning, DNACPR	0	3(75)	1(25)	0
Personal coping strategies	1(25)	1(25)	1(25)	1(25)
Reflective practice	1(25)	0	0	3(75)
Interdisciplinary teamwork	1(25)	0	2(50)	1(25)
Models of care: curative, palliative, disease-modifying	0	1(25)	1(25)	2(50)

### Clinical contact with palliative care providers

Table 2 provides an overview of clinical contact with palliative care providers, including general practice and aged care. Second-year students at one university are introduced to palliative care by working in residential aged care facilities as assistant caregivers for up to 1 week, with additional time for preparation and guided reflection afterwards. Contact with specialist palliative care providers is limited. All conveners reported that students visit a hospice for at least one half-day during their training, and one school offers an optional two-week placement for trainee interns as part of a community attachment. Elective hospice experiences are available for senior students in two locations, although only one student can be accommodated at a time. Experience in hospice day units is limited and depends on whether this service is available in the student’s location. There is very little exposure to hospital palliative care services and no opportunities for students to observe palliative care outpatient clinics. Most clinical exposure (outside the hospital setting) occurs in general practice settings where interaction with people with PEOLC needs occurs opportunistically.

**Table 2:** Clinical contact with palliative care providers

	None	Up to half day (%)	Up to 1 day(%)	1 day to 1 week (%)	> 1 week (%)	Repeated activity (%)
Hospice IPU	0	3 (75)	0	1 (25)	0	0
Hospice community team	1 (25)	2 (50)	1 (25)	0	0	0
Hospice day unit	3 (75)	0	1 (25)	0	0	0
PC outpatient clinic	4 (100)	0	0	0	0	0
Hospital PC service	2 (50)	2 (50)	0	0	0	0
Residential aged care	3 (75)	0	0	1(25)	0	0
PC patients in GP setting	1 (25)	0	2 (50)	0	0	1 (25)
PC elective	2 (50)	0	0	0	2 (50)	0

*PC* Palliative Care, *GP* General Practice

### Assessment

Formal assessment of learning in PEOLC was carried out on three campuses at the time of the survey. Table 3 shows that multiple-choice questions and reflective essays are the favoured approaches to assessment. Observed Structured Clinical Examinations (OSCE) involving PEOLC scenarios are used for summative assessment on one campus, which includes the use of opioids and explaining palliative care to a patient. Guided reflection following a filmed simulation of a breaking bad news scenario was reported by one convener to assess communication skills. Case presentations of a person with PEOLC needs was reported by one convener, although this only occurs opportunistically. Clinical supervisor feedback was limited to the school that provides a longer hospice attachment, which allows for more informed feedback about student competencies in PEOLC. Observed history and examination of a palliative care patient and extended multiple-choice questions were not reported at all.

**Table 3:**
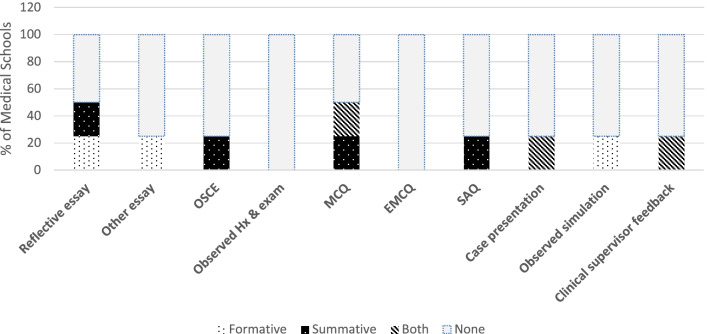
Assessment

### Barriers to development

The main barriers to development included pressure on curriculum time, lack of clinical attachments in palliative care, lack of assessment and lack of teaching staff. A further issue identified by one convener was a lack of time which limited capacity to develop their teaching. No conveners reported a lack of leadership or support for PEOLC teaching. All felt their colleagues were enthusiastic about PEOLC teaching, which indicates a good level of faculty support, despite these barriers.

## Discussion

This article reports the findings of the first national survey of formal undergraduate teaching in PEOLC at NZ medical schools. The results indicate there is enthusiasm and commitment for PEOLC teaching, which is reflected in the recent development of a national undergraduate medicine PEOLC curriculum and implementation framework. While PEOLC is well represented with more than 40 hours of formal teaching at three of the four campuses, the extent to which learning outcomes and objectives are addressed depends on the availability of space in the curriculum and the availability of teaching staff, which varies between campuses. Assessment processes could also be expanded and standardised across campuses, which could provide useful information about student learning and data for further curriculum development. However, the lack of academic appointments in palliative care limits what can be achieved without further investment and prioritisation.

Similar issues have been found in the undergraduate nursing curriculum in New Zealand where PEOLC teaching is less well represented. Nursing schools report the same lack of clinical placements with specialist palliative care providers and minimal formal assessment in PEOLC [32]. A new national undergraduate nursing curriculum is currently under development by Te Pukenga (The NZ Institute of Skills and Technology), which provides an ideal opportunity to increase PEOLC teaching content and assessment, and also creates opportunities for interprofessional collaboration between nursing and medicine.

The European Association of Palliative Care (EAPC) EDUPALL undergraduate palliative medicine curriculum provides a useful resource for curriculum development and recommends 72 hours of formal PEOLC teaching [33]. Despite recent progress, there is still a way to go before this target is met in New Zealand. Although adding new content to an already overcrowded course is challenging, the curriculum must reflect national health priorities, which include PEOLC [28]. Integrating teaching into existing modules and departmental teaching is one way of adding content without overstretching the curriculum and is the preferred approach in NZ medical schools, combined with dedicated block teaching to consolidate key concepts [33]. Organising teaching in this way creates opportunities for collaboration, which reflects the multidisciplinary nature of palliative care and teamwork. However, integration may come at a cost to visibility unless clear links are made with related learning and learning objectives are clearly defined and supported by supervised clinical encounters with palliative care patients, whānau and healthcare providers. Online learning platforms also serve as a useful resource for independent inquiry without the need for additional curriculum time. Web-based resources such as the New Zealand Health Quality and Safety Commission (hqsc.govt.nz), PCC4U (pcc4u.org.au), e-ELCA (Learning Path for Medical Students) (https://www.e-lfh.org.uk) provide consistent learning messages and optimise face to face discussion using a flipped classroom approach. Resources for the healthcare workforce, such as End of Life Essentials (https://www.endoflifeessentials.com.au) could also be used for further learning.

Holistic, person-centered care that upholds the values of compassion, empathy and respect underpin the delivery of palliative care in New Zealand (www.hospice.org.nz) [34]. This is reflected in the Māori model of health Te Whare Tapa Whā, developed by Professor Mason Durie in 1998 [35] which is integrated throughout formal teaching, although there is room for further development (see Table 1).

Assessment is an essential component of any curriculum and the results of this research indicate more formal PEOLC assessment should be included at all levels of the curriculum. A variety of methods should be used, including reflective reports following clinical encounters to help students reflect on their experiences, increase self-awareness and build emotional intelligence [36]. Given the collaborative relationship that exists between medical schools, assessments may be shared and the results used to provide useful data to inform curriculum development.

Without supervised clinical contact with people who have PEOLC needs, junior doctors will qualify without basic competencies in PEOLC. Unfortunately, clinical contact with specialist palliative care providers is limited by service capacity. Pedagogical strategies that enhance the development of PEOLC clinical competencies in generalist healthcare settings are therefore important, such as longitudinal cases with palliative care patients. People with PEOLC needs, such as chronic diseases and cancer can be found in most healthcare settings [2, 37]. Evidence suggests that non-specialist rotations that incorporate a palliative care approach are effective for learning, combined with structured activities such as simulation and small group discussions that address PEOLC concepts [38]. Continuing education for clinical staff to raise awareness and understanding about PEOLC, out of which palliative care champions emerge may also help facilitate student learning and provide valuable mentorship.

There are limitations to this research. The survey was not a formal audit of PEOLC teaching but was based on subjective reporting from module conveners. While there were only four conveners, their responses were representative and indicated that discussion with colleagues had occurred. However, subjective reporting means they may have over or under-estimated the perceived adequacy of the teaching they provide. Furthermore, PEOLC teaching is integrated throughout the curriculum so it can be difficult to accurately quantify in a complex course where this subject could be addressed in a variety of places. Similarly, exposure to people with PEOLC needs in generalist healthcare settings occurs opportunistically, so cannot be quantified. We recommend this survey is repeated in 2 years’ time to assess progress against the baseline provided by this research. Further research is also required to investigate the impact of PEOLC education on graduates’ self-efficacy and attitudes towards caring for people near the end of life.

## Conclusions

The increasing prevalence of people with multimorbidities associated with the aging population is placing extra pressure on health services in New Zealand and creating more demand for palliative care. Significant progress has been made towards developing undergraduate education in PEOLC in New Zealand medical schools. However, further investment is needed to support ongoing development to ensure graduates have the knowledge, skills and attitudes they need to provide safe, effective and compassionate care for patients and whānau (family) who are nearing the end of life, and to meet the needs of the population into the future.

## Data Availability

All data generated or analysed during this study are included in this published article.

## Abbreviations

PEOLC: Palliative and end of life care
NZ: New Zealand
